# Timing of early laparoscopic cholecystectomy for acute calculous cholecystitis: a meta-analysis of randomized clinical trials

**DOI:** 10.1186/s13017-021-00360-5

**Published:** 2021-03-25

**Authors:** Giuseppe Borzellino, Safi Khuri, Michele Pisano, Subhi Mansour, Niccolò Allievi, Luca Ansaloni, Yoram Kluger

**Affiliations:** 1 Department of General Surgery, University Hospital of Verona, Piazzale A. Stefani 1, 37128 Verona, Italy; 2 Department of General Surgery, Rambam Health Care Campus, Haifa, Israel; 3 1st Surgical Unit, Department of Emergency, ASST Papa Giovanni Hospital XXIII, Bergamo, Italy; 4 1st General Surgery Unit, University of Pavia, Pavia, Italy

**Keywords:** Acute cholecystitis, Laparoscopic cholecystectomy, Timing

## Abstract

**Background:**

Early cholecystectomy for acute cholecystitis has proved to reduce hospital length of stay but with no benefit in morbidity when compared to delayed surgery. However, in the literature, early timing refers to cholecystectomy performed up to 96 h of admission or up to 1 week of the onset of symptoms. Considering the natural history of acute cholecystitis, the analysis based on such a range of early timings may have missed a potential advantage that could be hypothesized with an early timing of cholecystectomy limited to the initial phase of the disease. The review aimed to explore the hypothesis that adopting immediate cholecystectomy performed within 24 h of admission as early timing could reduce post-operative complications when compared to delayed cholecystectomy.

**Methods:**

The literature search was conducted based on the Patient Intervention Comparison Outcome Study (PICOS) strategy. Randomized trials comparing post-operative complication rate after early and delayed cholecystectomy for acute cholecystitis were included. Studies were grouped based on the timing of cholecystectomy. The hypothesis that immediate cholecystectomy performed within 24 h of admission could reduce post-operative complications was explored by comparing early timing of cholecystectomy performed within and 24 h of admission and early timing of cholecystectomy performed over 24 h of admission both to delayed timing of cholecystectomy within a sub-group analysis. The literature finding allowed the performance of a second analysis in which early timing of cholecystectomy did not refer to admission but to the onset of symptoms.

**Results:**

Immediate cholecystectomy performed within 24 h of admission did not prove to reduce post-operative complications with relative risk (RR) of 1.89 and its 95% confidence interval (CI) [0.76; 4.71]. When the timing was based on the onset of symptoms, cholecystectomy performed within 72 h of symptoms was found to significantly reduce post-operative complications compared to delayed cholecystectomy with RR = 0.60 [95% CI 0.39;0.92].

**Conclusion:**

The present study failed to confirm the hypothesis that immediate cholecystectomy performed within 24 h of admission may reduce post- operative complications unless surgery could be performed within 72 h of the onset of symptoms.

## Background

Early timing of laparoscopic cholecystectomy is recommended for the treatment of acute cholecystitis since the current literature reports a shorter hospital stay after early cholecystectomy compared to delayed cholecystectomy [1, 2]. However, none of the published meta-analyses on the timing of cholecystectomy have reported any difference in morbidity between the two timings [3–10]. It should be emphasized that the definition of early cholecystectomy reported in the reviews was heterogeneous referring to intervention performed up to 96 h following the admission or up to 1 week following the onset of symptoms [3–10]. By considering such range of early timings within their analysis, the reviews did not allow to assess whether there may be an early timing that may influence outcomes and after which cholecystectomy should be considered no more as early but as delayed.

Based on the general pathology timing, acute inflammation tends to resolve after 72 h of the onset of an inflammatory process and let chronic inflammation take place [11, 12]. In patients with acute cholecystitis, all would therefore theoretically be expected to suffer local and systemic inflammation within 72 h of the onset of symptoms, but local and systemic changes may be, after this time, unpredictable for each patient. A rate of acute cholecystitis may be resolving and a rate may not be improving or even worsening as observed in delayed surgery strategy. As a result, once symptoms have been lasting for more than 72 h, any comparison between any different early and delayed timings could be more balanced. Moreover, having found no difference in morbidity may indicate that the compared early and delayed cholecystectomy within meta-analyses had an overlying risk of complications. It can be supposed therefore that performing cholecystectomy during the initial phase of the disease may prevent the complications related to the on-going cholecystitis at the time of surgery, especially for those evolving into a severe form for which post-operative complications are increased [13].

The Japanese Guidelines TG13 [14] recommended early cholecystectomy to be performed soon after the admission when less than 72 h have passed since the onset of symptoms but the recommendation was mainly based on expert opinion and setting early cholecystectomy within 72 h of symptoms is still controversial. In randomizing cholecystectomy before and after 72 h of symptoms during the same admission [15], a recent small trial found a difference in one oxidative stress marker but no correlation with post-operative complications. The latest Japanese guidelines TG18 [1] confirm the recommendation to perform early cholecystectomy within 72 h of symptoms but recommend also to perform early cholecystectomy as soon as possible even after this time, regardless of the onset of symptoms. According to Gull et al. [16], immediate cholecystectomy should not refer to the onset of symptoms but to admission since referring the timing of early cholecystectomy to the onset of symptoms is not always feasible because of the subjective perception of signs and symptoms. In their trial, the authors compared immediate cholecystectomy performed within 24 h of admission to delayed cholecystectomy and found a significant reduction in overall morbidity rate. However, the trial actually compared an early timing to a delayed timing and did not provide any information on immediate timing compared to other early timings. Moreover, in this trial, overall morbidity included complications occurred while waiting cholecystectomy the rate of which is expected to be greater in the delayed group and above all are poorly related to the early timing.

The clinical question on when early cholecystectomy should be performed has therefore not yet been answered. For the purpose, cholecystectomy performed within 24 h of admission appears to be the most appropriate option to evaluate the effectiveness of an immediate timing in clinical practice, moreover post-operative complications appear more appropriate than overall morbidity when exploring the role of immediate cholecystectomy within the early timing.

This study aimed to explore, through a review of the literature, the hypothesis that in patients with acute cholecystitis fit for urgent surgery, adopting immediate cholecystectomy performed within 24 h of admission as early timing could reduce post-operative complications when compared to delayed cholecystectomy.

## Methods

### Protocol and registration

A protocol reporting the methods of the meta-analysis was published [17] and registered on the PROSPERO database with number: CRD42020149600. The meta-analysis was performed according to the Preferred Reporting Items for Systematic Reviews and Meta-Analyses (PRISMA) statement [18].

### Data sources

Two authors independently conducted a literature search, according to the Patient Intervention Comparison Outcome Study (PICOS) strategy. Subject headings and text words were used to identify randomized studies that included patients with acute cholecystitis submitted to laparoscopic cholecystectomy at different timings and that reported post-operative complications (Table 1). Neither date limits nor language limits were imposed. A translation was foreseen in case of a language not known to any of the authors.

**Table 1:** Detailed search strategy on the databases

Database	Search strategy	Found articles
PubMed	((((((((cholecystitis[MeSH Terms]) OR acute cholecystitis[MeSH Terms]) OR cholecystitis, acute[MeSH Terms])) AND ((((laparoscop*) OR celioscop*) OR coelioscop*) OR peritoneoscop*)) AND ((cholecystectomy) OR cholecystectomies)) AND (((((immediate) OR early) OR urgent) OR delayed) OR timing)) AND (((morbidit*) OR complication*) OR post-operative)) AND random*	66
Cochrane Library	acute cholecystitis and cholecystectomy and randomized (publication type)	99
Embase	acute AND cholecystitis AND cholecystectomy AND [article]/lim AND [randomized controlled trial]/lim	107
ClinicalTrials.gov	acute cholecystitis and cholecystectomy and interventional studies and (terminated or completed)	14
PakiMedNet	acute cholecystitis randomized	8

The literature search was conducted on PubMed, completed by consulting the Cochrane Library, Embase, and ClinicalTrials.gov and by reviewing the references of the found reviews and meta-analyses. Based on the review findings, the search was extended to Google Scholar and PakiMedNet database. Unpublished studies and data from presentations to Congress were not considered.

### Studies selection

Two authors independently selected the studies while a third author was in charge in case of disagreement between the two authors. Studies were included in the current analysis only if they were randomized trials comparing different timings of laparoscopic cholecystectomy, in which the criteria for the diagnosis of acute cholecystitis, the population study, timing for surgery, and data on post-operative complications were reported.

Studies were excluded if the timing was defined using imprecise language without an exact numerical timing for the intervention, the population study was not defined, any clinically relevant categories of patients were excluded or patients with diseases other than acute cholecystitis were included.

### Data collection

Data were independently collected by two authors and reported in a pre-prepared sheet. Data of studies comparing early timing to delayed timing of cholecystectomy were collected. The main outcome was the post-operative complication rate. Data were grouped based on three different timings of cholecystectomy: early timing within 24 h of admission defining immediate cholecystectomy, early timing over 24 h of admission and delayed timing referring to elective cholecystectomy performed after medical treatment. The hypothesis, that immediate cholecystectomy could reduce post-operative complications was therefore explored by comparing pooled data on early timing within 24 h of admission versus delayed timing of cholecystectomy and pooled data on early timing over 24 h admission versus delayed timing of cholecystectomy within a sub-group analysis.

The findings of studies, the timing of which did not refer to admission but to the onset of symptoms prompted to anticipate a second analysis that in the protocol was foreseen in the sensitivity analysis. These studies appeared to be of interest since they reported data on cholecystectomy performed within 72 h of the onset of symptoms, allowing a more suited analysis to the physiopathological hypothesis. As a result, the studies were included and data collected for a potential second analysis in which early timing within 72 h of symptoms and early timing over 72 h of symptoms were both compared to delayed timing of cholecystectomy within a subgroup analysis.

All complications were attributed to the initial assigned group with no distinction between complications occurred after cholecystectomy completed by laparoscopy and after cholecystectomy that required a conversion to laparotomy. Three other secondary outcomes were registered: bile duct injury, conversion and mortality. All reported bile duct injuries were registered without distinguishing those diagnosed both intra-operatively and immediately repaired from those diagnosed post-operatively. In any case, bile duct injuries were analyzed separately while post-operative bile leaks were considered within post-operative complications. All conversions were registered even when it was reported the reason was an intra-operative diagnosis of bile duct injury.

### Risk of bias in individual studies

Two authors independently assessed the risk of bias. The quality assessment focused on the risk of bias arising from the randomization process, allocation concealment, blinding, missing outcome data, the measurement of the outcome, and selective reporting. Three different levels of risk (low, uncertain and high) were incorporated according to the findings of the risk of bias assessment.

### Statistical methods

Since studies were all randomized and selected based on defined criteria, neither clinical nor methodological heterogeneities were expected. The relative risk (RR) and its 95% confidence interval (95% CI) were calculated adopting a fixed-effect model [19]. Heterogeneity was estimated with the chi-squared test and the I^2^ statistic and was excluded when the chi-squared was not significant and I^2^ < 25% [20, 21]. For sub-group difference analysis a chi-squared test was performed. The level of significance was set at 0.05. The meta-analysis was conducted using ReviewManager (RevMan) software (version 5.3) [22].

### Risk of bias across studies

The quality of evidence was evaluated according to the Grading of Recommendations, Assessment, Development and Evaluations (GRADE) framework [23]. The five domains that can lower the certainty of a body of evidence were considered as follows: risk of bias, inconsistency across studies, indirectness of studies, imprecision of studies, and publication bias. The rating up of the evidence was considered in case of a large effect. Publication bias was explored with a funnel plot by using the asymmetry of trial size against treatment effect to assess this bias [24, 25].

### Sensitivity analysis

The fixed-effect model was compared to the random-effects model using the DerSimonian Laird method [26]. A further analysis was performed based on the exclusion of those studies the inclusion of which required a discussion. The role of excluded studies because of incomplete information about the exact timing of cholecystectomy or methodological aspect of the research was explored by including each of them in the immediate or delayed group according to the indicated or best estimate of the cholecystectomy timing.

## Results

### Study selection

**Fig. 1:**
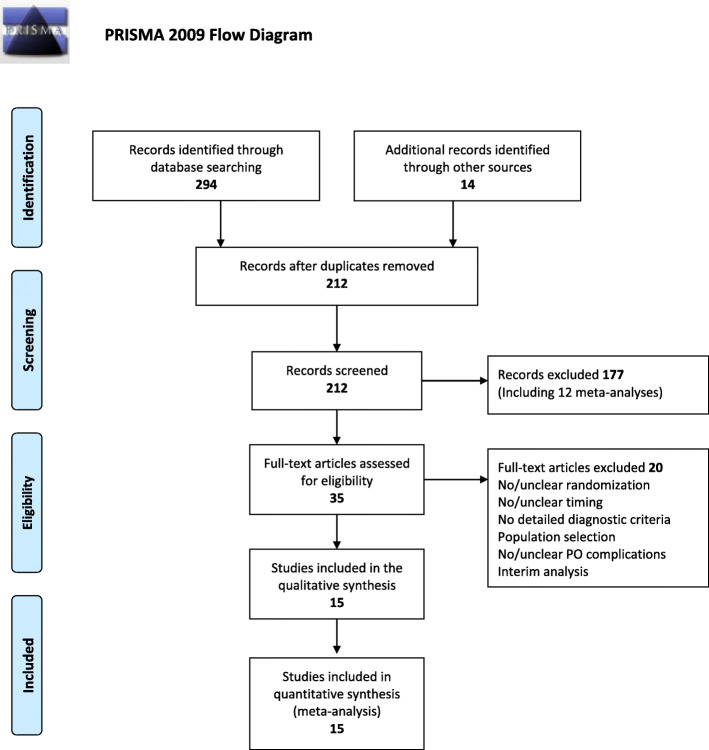
Literature search flow chart

As reported in Fig. 1, there were 294 studies found from the five databases, with another 14 articles from other sources. After reading the titles and abstracts and eliminating duplicates, 35 articles were selected for full-text reading, of which 15 were included in the analysis [15, 27–40].

### Study characteristics

In total, three studies compared early timing within 24 h of admission to delayed timing of cholecystectomy [27–29] while six studies reported a comparison between early timing over 24 h of admission (from 48 to 72 h) and delayed timing of cholecystectomy [30–35]. However, the studies on early timing within 24 h of admission included in the early group patients with symptoms persisting up to 1 week [27] or up to 96 h [28], while in one study it was not possible to assess the exact timing related to the onset of symptoms [29]. Moreover, six additional studies that compared early timing within 72 h of symptoms to delayed timing of cholecystectomy were selected for a second analysis [15, 36–40]. The inclusion of one study in which patients were randomized to be operated on before and after 72 h of symptoms required discussion [37]. As all patients received medical treatment, the timing of 72 h after the symptoms was considered to be as delayed. The characteristics and the results of the studies reporting data on early timing of cholecystectomy performed within 24 h of admission and within 72 h of symptoms are summarized in Table 2.

**Table 2:** Characteristics of studies that reported post-operative complications in comparing immediate cholecystectomy performed within 24 h from admission or within 72 h from on the onset of symptoms, compared to delayed cholecystectomy

Authors	Year	Patients		Timing		Inclusion criteria	Exclusion criteria	Pathology confirmation	Post-operative complications		*P* value
		Early	Delayed	Early	Delayed				Early	Delayed	
Lai [27]	1998	53	51	< 24 h from admission	6–8 weeks	Clinical Laboratory Ultrasound	CBD stone complications Previous surgery High risk for surgery Symptoms for more than 7 days	Yes	5	3	0.8
Kolla [28]	2014	20	20	< 24 h from admission	6–12 weeks	Clinical Laboratory Ultrasound HIDA	CBD stone complications Previous surgery High risk for surgery Symptoms for more than 96 h	No	2	3	NR
Ozkardes [29]	2014	30	30	< 24 h from admission	6–8 weeks	Clinical Laboratory Ultrasound	CBD stone complications Previous surgery High risk for surgery	No	5	0	NR
Saber [36]	2014	60	60	< 72 h from symptoms	6–8 weeks	Clinical Laboratory Ultrasound	NR	No	6	4	NR
Jan [37]	2016	50	50	< 72 h from symptoms	> 72 h from symptoms Up to 7 days	Clinical Laboratory Ultrasound	CBD stone complications Previous surgery High risk for surgery Gallbladder malignancy Acalculus cholecystitis Symptoms for more than 7 days	No	1	3	NR
Rajock [38]	2016	31	31	< 72 h from symptoms	6–8 weeks	Clinical Laboratory Ultrasound	NR	Yes	3	8	NR
Arslan Onuk [15]	2019	32	32	< 72 h from symptoms	> 72 h from symptoms Up to 6 days after initial treatment	Clinical Laboratory Ultrasound	CBD stone complications Previous surgery Perforation Sepsis Pregnancy Immunosuppression	Yes	4	9	0.12
Arafa [39]	2019	74	74	< 72 h from symptoms	6–12 weeks	Clinical Laboratory Ultrasound	CBD stone complications Previous surgery High risk for surgery Free biliary perforation Pregnancy Decompensated cirrhosis No consent available	Yes	10	17	NR
El Kordy [40]	2019	20	20	< 72 h from symptoms	6–8 weeks	Clinical Laboratory Ultrasound	NR	No	4	6	NR

In total, 20 studies were excluded for the following reasons: the absence of or uncertainty about randomization [41–45], lack of reporting or uncertainty about timing [41, 46–50], lack of reporting or uncertainty about diagnostic criteria for acute cholecystitis [41, 45, 46, 51–54], limitation of the population study to the first episode and inclusion of biliary colic cases [53], inclusion of only patients with symptoms persisting for more than 72 h [50], exclusion of elderly patients [54], lack of reporting post-operative complications or lack of specifying whether the reported complications were primarily post-operative [16, 41, 48, 49, 55–58], or because the repoting of interim results of a randomized study [59].

### Risk of bias within studies

Only five studies reported a computer-generated randomization sequence allowing a low risk of allocation concealment bias [27, 28, 33, 36, 39], seven studies reported an odd-even simple randomization method [15, 29, 30, 34, 35, 37, 40] and three studies did not report data on randomization method [31, 32, 38], with, as a result, an uncertain or high risk of selection bias. None of the studies reported any blinding. While the absence of information on blinding of operators and patients could be considered to be at low risk of bias (being hardly feasible in surgical trials), the absence of declared blinding of the outcome assessment may be of concern and the risk should be considered as uncertain [60]. The risk of bias related to the missing outcome data, the measurement of the outcome and selective reporting was considered as low for all the studies.

### Synthesis of results

**Fig. 2:**
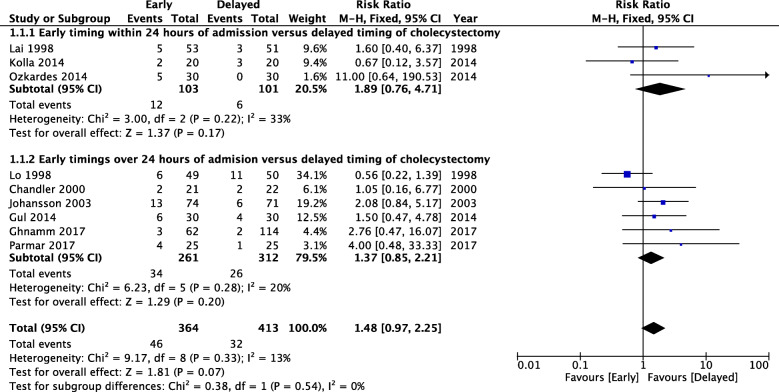
Sub-group comparisons on post-operative complications based on timing related to time from admission: early timing of cholecystectomy within 24 h of admission defining immediate cholecystectomy and early timing of cholecystectomy over 24 h admission, both compared to delayed cholecystectomy

The comparison of pooled data on immediate versus delayed cholecystectomy failed to find any difference. The risk of post-operative complications was not significantly different when early timing within 24 h of admission was compared to delayed timing of cholecystectomy with RR = 1.89 [95% CI 0.76; 4.71]. Also, no difference was found by comparing early timing over 24 h of admission to delayed timing of cholecystectomy with RR = 1.37 [95% CI 0.85; 2.21]. Heterogeneity could not be excluded in the first comparison. The test for differences between sub-groups did not provide a significant result with *p* = 0.54 (Fig. 2).

**Fig. 3:**
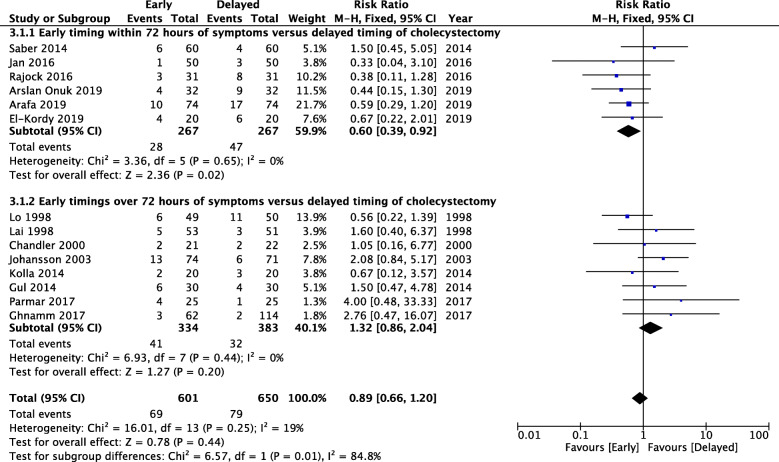
Sub-group comparison on post-operative complications based on timing related to time from the onset of symptoms: early cholecystectomy performed within 72 h of symptoms and early timing of cholecystectomy all over 72 h of symptoms, both compared to delayed cholecystectomy

Within the second analysis, pooling data showed a statistically significant reduction in the rate of post-operative complications with RR = 0.60 [95% CI 0.39; 0.92] when early timing within 72 h of symptoms was compared to delayed timing of cholecystectomy. No heterogeneity was found. Among the included studies, eight of them reported a comparison between early cholecystectomy the timing of which was in all over 72 h of symptoms, and delayed cholecystectomy [28–35]. This finding allowed a sub-groups analysis based on the two different early timings, before and after 72 h of symptoms, both compared to delayed cholecystectomy. Pooled data from these studies did not show a significant difference between early timing over 72 h of symptoms and delayed cholecystectomy with RR = 1.32 [95% CI 0.86; 2.04]. No heterogeneity was found. Moreover, the comparison between the two sub-groups showed a statistically significant difference with *p* = 0.01, giving strength to the results concerning cholecystectomy performed within 72 h of symptoms (Fig. 3). The details of the post-operative complications related to this sub-group comparisons are reported in Table 3.

**Table 3:** Detailed post-operative complications reported within the studies included in the sub-group analysis that considered early timing related to the onset of symptoms

	Early timing of cholecystectomy within 72 h of symptoms	Delayed cholecystectomy
Bile leak	5	3
Ileus	1	6
Intra-abdominal collection	2	5
Intra-abdominal bleeding	1	3
Wound and parietal hematoma or bleeding	1	2
Wound infection	18	27
Retained stones	0	1
**Total**	**28**	**47**
	Early timing of cholecystectomy over 72 h of symptoms	Delayed cholecystectomy
Bile leak	11	4
Ileus	0	2
Intra-abdominal collection	5	7
Intra-abdominal bleeding	0	1
Wound infection	10	8
Retained stones	3	0
Other infections	9	6
Respiratory complications	3	3
Atrial fibrillation	0	1
**Total**	**41**	**32**

Overall ten studies reported data on biliary injury [27, 28, 30–35, 37, 39]. Among the studies that focused on immediate cholecystectomy performed within 24 h of admission compared to delayed cholecystectomy, two of them reported data on biliary injury [27, 28] but RR was not estimable because, one study reported no biliary injury [27] and one only one case in the immediate cholecystectomy group [28]. When considering studies that compared early cholecystectomy performed within 72 h of symptoms to delayed cholecystectomy, two studies reported data on biliary injury [37, 39], no significant difference was found with RR = 0.23 [95% CI 0.04; 1.34].

All included studies reported data on conversion. The analysis based on studies that compared immediate cholecystectomy performed within 24 h of admission to delayed cholecystectomy [27–29] reported no significant difference in conversion with RR = 1.21 [95% CI 0.68; 2.17] and *I*^2^ = 15%. When considering studies for which timing referred to the onset of symptoms [15, 36–40], cholecystectomy performed within 72 h of symptoms significantly reduced the rate of conversion compared to delayed cholecystectomy with RR = 0.53 [95% CI 0.32; 0.89] and *I*^2^ = 0%.

Reported mortality was very low and data were not sufficient to perform a meta-analysis on this variable.

### Quality of evidence

As the present meta-analysis only included randomized studies, the level of evidence should first be considered high according to the GRADE rule. The only domain that should be considered in rating down the quality of evidence has been the potential risk of bias. None of the dedicated domains could allow the level of evidence to be rated up. The risk of bias involved not only the method of randomization, allocation concealment and blinding but also the lack of pathological confirmation of a diagnosis of acute cholecystitis. While all cases are expected to be non-acute at the time of surgery in the delayed group, an unknown rate of non-acute cases in the immediate group because of a diagnostic error may be source of an overestimation of the benefit of immediate cholecystectomy.

**Fig. 4:**
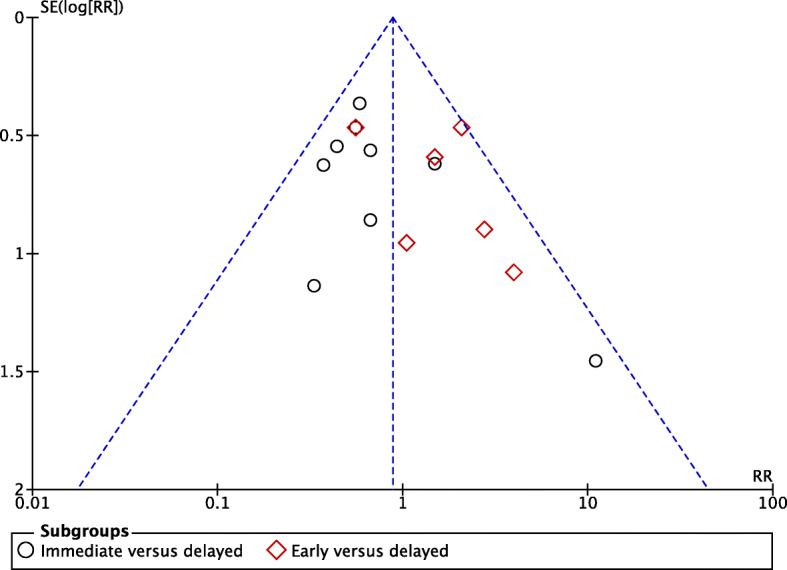
Funnel plot assessing the risk for potential publication bias

A large magnitude of the effect was not found, the dose-response gradient was not applicable and no potential residual confounders would decrease the magnitude of the effect. The funnel plot shown in Fig. 4 illustrates the low risk for potential publication bias in this study. Overall, the quality of evidence of this meta-analysis should be considered moderate.

### Sensitivity analysis

The sensitivity analysis performed by applying the random-effects model revealed similar results compared to the fixed-effect model, for both immediate cholecystectomy performed within 24 h of admission and for cholecystectomy performed within 72 h of symptoms. About early timing within 24 h of admission versus delayed timing of cholecystectomy RR = 1.61 [95% CI 0.44; 5.85] with *I*^2^ = 33%; about early timing over 24 h of admission versus delayed timing of cholecystectomy RR = 1.38 [95% CI 0.77; 2.46] with *I*^2^ = 20%; *p* = 0.83 for the sub-group comparison. About early timing within 72 h of symptoms versus delayed timing of cholecystectomy RR = 0.60 [95% CI 0.39; 0.93] with *I*^2^ = 0%; about early cholecystectomy over 72 h of symptoms versus delayed cholecystectomy RR = 1.29 [95% CI 0.82; 2.03] with *I*^2^ = 0%; *p* = 0.02 for the sub-group comparison.

According to the literature finding and selected studies, no other sensitivity analysis was feasible for comparisons based on immediate cholecystectomy performed within 24 h of admission. The two further planned sensitivity analyses were therefore limited to the comparisons based on cholecystectomy performed within 72 h of symptoms. By excluding the study which was a matter of discussion [37], sensitivity analysis confirmed the results on the risk of post-operative complications: RR = 0.61 [95% CI 0.40; 0.95] with *I*^2^ = 0% for cholecystectomy performed within 72 h of symptoms compared to delayed cholecystectomy; RR = 1.32 [95% CI 0.86; 2.04] with *I*^2^ = 0% when other early timings were compared to delayed cholecystectomy; *p* = 0.01 for the sub-group comparison.

When investigating the effect of the inclusion of studies that had been omitted because of incomplete information regarding the exact timing of cholecystectomy, the absence of reported criteria for the diagnosis of acute cholecystitis, or other methodological aspects of the studies, the sensitivity analysis confirmed the results of the main comparison: RR = 0.60 [95% CI 0.39; 0.92] with *I*^2^ = 0% for cholecystectomy performed within 72 h of symptoms compared to delayed cholecystectomy; RR = 1.10 [95% CI 0.83; 1.46] with *I*^2^ = 7% when other early timings were compared to delayed cholecystectomy; *p* = 0.02 for the sub-group comparison.

## Discussion

The present review failed to confirm the hypothesis that immediate cholecystectomy performed within 24 h of admission may influence post-operative complications but a limited number of studies were found, which furthermore included patients with late presentation. However, an additional timing analysis based on the natural history of acute cholecystitis found that early timing within 72 h of symptoms reduced post-operative complications compared to delayed timing of cholecystectomy. Moreover, the finding that conversions were also reduced when adopting such timing gives strength to the hypothesis that cholecystectomy may be safer if performed, when possible, in the initial phase of acute cholecystitis.

The quality of evidence was rated as moderate because of the finding of a potential risk of selection bias. The risk was due to the method of randomization, allocation concealment, and blinding, as well as a lack of pathological confirmation of the diagnosis of acute cholecystitis. Among the selected studies on early timing of cholecystectomy within 24 h of admission or within 72 h of symptoms, only three reported a computer-generated randomization [27, 28, 39] while one did not report data on the randomization process [38] and four reported a simple randomization method with an uncertain or high allocation concealment risk [15, 29, 37, 40]. Globally, the level of risk related to the absence of blinding of operators and patients was considered being low while that related to the absence of blinding of the outcome assessment was uncertain [60]. Although the combined clinical, laboratory, and imaging criteria for acute cholecystitis have a low risk of diagnostic error [1], without a pathological confirmation of the diagnosis, which was reported only in one among the studies on immediate cholecystectomy performed within 24 h [27] and in three among the studies on early cholecystectomy performed within 72 h of symptoms [30, 38, 39], the inclusion of non-acute cases within the studies could not be excluded [2]. Randomization would equally distribute these patients in both arms but, while all patients are expected to be non-acute in the delayed group, a percentage of non-acute cases within the early group may be a cause of bias in estimating the effect of early timing of cholecystectomy. Moreover, the rate of confirmed severe cholecystitis in immediate cholecystectomy within 24 h of admission or within 72 h of symptoms compared to other timings is reported only in two studies [27, 30], not allowing to extrapolate any conclusive data on the rate of on-going acute cholecystitis according to the timing of surgery.

Signficant results on post-operative complications are limited to patients submitted to early surgery performed within 72 h of symptoms, but surgery is not feasible within such timing for all patients because of possible late presentation as reported in the selected studies [27, 28, 30–35]. Moreover, the data on the feasibility of surgery within such timing in the general population are limited. Among the studies on early timing within 72 h of symptoms [15, 36–40], only one study [15] reported the rate of excluded patients because of symptoms lasting for more than 72 h at admission, such rate was 54% of all the patients admitted for acute cholecystitis during the study period. Some reported exclusion criteria, such as patients with sepsis, severe cholecystitis with organ failure, perforation, or those at high surgical risk because of medical illness, were mandatory for ethical reasons. Nevertheless, only complications related to biliary migration in the common bile duct and previous surgery [15, 37, 39] as well as pregnancy [15] should be considered when defining the population to which the results of the present meta-analysis should be applied.

The present study focused on post-operative complications as the main outcome. For the purpose of the meta-analysis, both local as well as systemic complications whenever reported by the selected studies were considered. The main result of the present study has not confirmed the finding of the analysis reported within TG 18 guidelines [1] according to which complications were reduced in early cholecystectomy even when performed after 72 h of the onset of symptoms. The finding of TG 18 referred to a comparison reported within a meta-analysis [8], which was however limited to wound infections. Within the analysis comparing cholecystectomy performed within 72 h of symptoms to delayed cholecystectomy in the present study, wound infections were the most frequently reported post-operative complication but were not the only one that favoured early cholecystectomy [36–38, 40].

Perioperative or overall morbidity could have been considered for the evaluation of the timing of cholecystectomy, but such outcomes should include not only post-operative complications but also, when reported, intra-operative complications and complications while awaiting cholecystectomy. Intra-operative complications do not substantially change the course of a patient’s history unless they require conversion or involve bile duct injury. Both have been analyzed as secondary outcomes in the present review, with the finding that cholecystectomy performed within 72 h of symptoms reduced conversion when compared to delayed cholecystectomy, while no significant differences were found in instances of bile duct injury with low prevalence in both early and delayed cholecystectomy. Moreover, none of the selected studies reported which was the rate of complications while awaiting cholecystectomy.

Most studies focused on the failure rate of conservative treatment and re-admission for the recurrence of symptoms of acute cholecystitis while awaiting cholecystectomy [27, 28, 30–32, 34–36, 38, 46, 50, 51, 53]. These outcomes however concern the delayed cholecystectomy that is performed weeks after the acute phase of cholecystitis and have no interest when investigating different early timings of cholecystectomy. However, the risk of failure of conservative treatment and of re-admission for the recurrence of acute cholecystitis in delayed surgery, as well as the reduction in length of hospital reported in all the meta-analyses [3–10], the related cost reduction [16, 29, 34] and better quality of life [61] favoring early surgery, should be considered when deciding the timing of cholecystectomy for those patients whose symptoms were lasting for more than 72 h at the time of a feasible surgery.

## Conclusions

The present study failed to confirm the hypothesis that immediate cholecystectomy performed within 24 h of admission may reduce post-operative complications. However, the finding of studies in which timing did not refer to admission but to the onset of symptoms allows to suggest that immediate cholecystectomy should be preferred to delayed cholecystectomy when feasible within 72 h of the onset of symptoms. Once symptoms have persisted for more than 72 h at the time of a feasible surgery, others outcomes and risk factors should be considered when deciding the timing of cholecystectomy.

## Data Availability

The datasets used and/or analyzed during the current study are available from the corresponding author upon reasonable request.

## Abbreviations

PICOS: Patient Intervention Comparison Outcome Study
RR: Relative risk
CI: Confidence interval
PRISMA: Preferred Reporting Items for Systematic Reviews and Meta-Analyses
GRADE: Grading of Recommendations, Assessment, Development and Evaluations
